# *Nigella sativa* Extract Potentially Inhibited Methicillin Resistant *Staphylococcus aureus* Induced Infection in Rabbits: Potential Immunomodulatory and Growth Promoting Properties

**DOI:** 10.3390/ani12192635

**Published:** 2022-09-30

**Authors:** Gamal Abd Elmoneim Elmowalid, Adel Attia M. Ahmad, Marwa I. Abd El-Hamid, Doaa Ibrahim, Ali Wahdan, Amal S. A. El Oksh, Ahlam E. Yonis, Mohamed Abdelrazek Elkady, Tamer Ahmed Ismail, Adel Qlayel Alkhedaide, Shimaa S. Elnahriry

**Affiliations:** 1 Department of Microbiology, Faculty of Veterinary Medicine, Zagazig University, Zagazig 44511, Egypt; 2 Department of Nutrition and Clinical Nutrition, Faculty of Veterinary Medicine, Zagazig University, Zagazig 44511, Egypt; 3 Department of Bacteriology, Immunology and Mycology, Faculty of Veterinary Medicine, Suez Canal University, Ismailia 41522, Egypt; 4 Reference Laboratory for Quality Control of Poultry Production (RLQP), Department of Biotechnology, Animal Health Research Institute (AHRI), Sharkia Branch, Agriculture Research Center (ARC), Zagazig 44511, Egypt; 5 Reference Laboratory for Veterinary Quality Control of Poultry Production (RLQP), Department of Biotechnology, Animal Health Research Institute (AHRI), Damanhour Branch, Agriculture Research Center (ARC), Zagazig 44511, Egypt; 6 Department of Bacteriology, Animal Health Research Institute (AHRI), Mansura Branch, Agriculture Research Center (ARC), Mansoura 35511, Egypt; 7 Department of Clinical Laboratory Sciences, Turabah University College, Taif University, P.O. Box 11099, Taif 21944, Saudi Arabia; 8 Department of Bacteriology, Mycology and Immunology, Faculty of Veterinary Medicine, University of Sadat City, Sadat City 32897, Egypt

**Keywords:** *Nigella sativa* extract, MRSA, rabbits, antimicrobials, growth promotion, immunomodulation

## Abstract

**Simple Summary:**

The high incidence of stress-associated diseases post rabbit weaning results in great losses threatening the rabbit industry. The increasing emergence of multidrug resistant (MDR) methicillin-resistant *Staphylococcus aureus* (MRSA) causes life threatening infections worldwide. Thus, the necessity to look inward for natural alternative treatments is now compelling. In this perspective, *Nigella sativa* extract (NSE) could serve as an effective antibiotic alternate source against MRSA. Herein, NSE was found to possess iin vitro antimicrobial activities against MRSA clinical isolates. Moreover, the synergistic activity between NSE and other antimicrobials was employed to overcome the MRSA resistance. Our findings added new insights for application of NSE in diets of growing rabbits as a growth promoting and an immunostimulant agent, which in turn reduced the high risk associated MRSA infections in growing rabbits.

**Abstract:**

Weaning is the most crucial period associated with increased stress and susceptibility to diseases in rabbits. Methicillin-resistant *Staphylococcus aureus* (MRSA), a historic emergent pathogen related to post weaning stressors, adversely affects rabbit’s growth rate and productive cycle. Since MRSA is rapidly evolving antibiotics resistance, natural products are desperately required to tackle the public health threats posed by antimicrobial resistance. Thus, this study aimed to screen the iin vitro antibacterial activity of *Nigella sativa* extract (NSE) and its interactions with antibiotics against MRSA isolates. Moreover, 200 weaned rabbits were divided into 4 groups to investigate the iin vivo superiority of NSE graded levels towards growth performance, tight junction integrity, immune responsiveness and resistance against MRSA. Herein, NSE showed promising antimicrobial activities against MRSA isolates from animal (77.8%) and human (64.3%) origins. Additionally, MRSA isolates exposed to NSE became sensitive to all antimicrobials to which they were previously resistant. Our results described that the growth-promoting functions of NSE, especially at higher levels, were supported by elevated activities of digestive linked enzymes. Post-NSE feeding, rabbits’ sera mediated bactericidal activities against MRSA. Notably, upregulated expression of occludin, *CLDN-1*, *MUC-2* and *JAM-2* genes was noted post NSE supplementation with maximum transcriptional levels in 500 mg/kg NSE fed group. Our data described that NSE constitutively motivated rabbits’ immune responses and protected them against MRSA-induced experimental infection. Our results suggest the antimicrobial, growth stimulating and immunomodulation activities of NSE to maximize the capability of rabbits for disease response.

## 1. Introduction

Rabbits’ production has been increased in developing countries due to its high economic and health importance for humans. Weaning is the most critical issue during rabbits’ life as it greatly affects their health and growth performance throughout the fattening period. For many years, antibiotics and other chemotherapeutic agents used to control several bacterial diseases associated with weaning period accelerate the evolution of antimicrobial resistance [1,2,3,4]. *Staphylococcus aureus* (*S. aureus*) is one of the most serious bacterial pathogens facing rabbits under stressful conditions and causing life-threatening infections in both animals and humans. Besides its pathogenicity and virulence potential, the development and/or acquisition of resistance to antimicrobials is of foremost importance. Occurrence of strains with a multidrug resistance (MDR) phenotype is common in staphylococcal species; particularly methicillin-resistant *S. aureus* (MRSA) strains those have become a major problem in healthcare settings and in the community [5]. They have common resistance to macrolides and tetracyclines in addition to developing resistance to aminoglycosides and quinolones. MRSA strains are generally associated with increased burden regarding therapeutics and higher mortality rates than methicillin-susceptible *S. aureus* [6,7]. Therefore, natural bioactive antimicrobials are strictly required as immune boosters for rabbits aiming at higher production efficiency. Moreover, World Health Organization recommendations suggested the need to have research alternatives to antibiotics to overcome the drawbacks of antibiotics abuse and microbial resistance [8,9] such as in the case of MRSA, which became endemic worldwide [10,11,12,13,14,15].

One of the natural alternatives to synthetic antibiotics is the seeds of black cumin (*Nigella sativa* L.). *Nigella sativa* (*N. sativa*) seeds contain bioactive constituents of isoquinoline alkaloids, including thymoquinone (TQ), thymohydroquinone (THQ) and di-thymoquinone, which have been reported to have many biological and immunological activities. Thymoquinone represents 30–48% of the seeds and it has a broad-spectrum antibacterial activity, especially against Gram-positive bacteria [16,17,18,19]. The antimicrobial activities of *N. sativa* crude oil extracted by organic solvents and purified bioactive substances, including TQ and THQ extracted by super-critical carbon dioxide, were evaluated iin vitro [17,20,21,22] and they were promising as alternative antimicrobials in a dose-dependent manner against Gram-positive *S. aureus* and Gram-negative *Escherichia coli* (*E. coli*) bacteria [23]. Clinical studies showed that *N. sativa* extract (NSE) has a great potential as an effective antimicrobial against bacteria isolated from skin infections with a special concern against *S. aureus*. A synergistic activity between the extracted oil and other antimicrobials was exerted to overcome resistant [22]. The antibacterial activities of both TQ and THQ could be potentiated by antibiotics, especially against *S. aureus* [24]. Because of the scarcity of studies demonstrating the antibacterial activities of *N. sativa* seeds or its active components, the present study had been carried out to explore the iin vitro inhibitory activities of NSE alone and in combination with different antibiotics against MRSA isolates. The ultimate aim of the current work was to investigate the iin vivo potential growth-promoting, immunostimulant and antibacterial activities of NSE to fight against MRSA clinical isolates in rabbits.

## 2. Materials and Methods

### 2.1. Samples Collection and Isolation and Characterization of MRSA

Fifty-two samples were collected from human subjects including pus (n = 15), sputum (n = 12), urine (n = 10), diabetic feet samples (n = 5), cerebrospinal fluid (n = 4) blood (n = 3) and burn swabs (n = 3) from public and private clinical laboratories at Sharkia Governorate, Egypt. Another 15 samples were collected from animal sources: nine cows’ milk and six meat products from retail outlets in Zagazig city, Sharkia, Egypt. All the steps of samples collection, processing and bacterial isolation and identification were done under strict aseptic conditions. All the study samples were processed and then inoculated onto Baird-Parker and Mannitol Salt Agar (Oxoid, Basingstoke, Hampshire, UK) plates and consequently, the plates were incubated for 24–48 h at 37 °C. Presumptive *S. aureus* isolates were phenotypically identified via standard bacteriological procedures basing on fermentation on Mannitol Salt Agar, β-hemolysis onto blood agar (Oxoid, Basingstoke, Hampshire, England, UK), formation of golden yellow pigments on milk agar, appearance of Gram-positive grape-like clusters and biochemical tests’ results such as positive catalase reaction and the ability to coagulate rabbit plasma [25,26]. Furthermore, biotyping of *S. aureus* isolates was carried out using API 20 Staph identification kit (BioMerieux, Marcy l’Etoile, France). Finally, the identities of putative isolates were defined genetically as *S. aureus* via PCR amplification of the species-specific *nuc* gene and as MRSA through PCR detection of *mec*A gene according to the cycling conditions described elsewhere [15,27,28].

### 2.2. Preparation of NSE

*Nigella sativa* seeds were purchased from Egyptian markets, then washed and dried. Ether aqueous extract was prepared by soaking 125 g of the dried *N. sativa* seeds into 250 mL of diethyl ether solution and then the mixture was kept overnight at room temperature. Consequently, it was filtered in sterile laminar-flow hood using Whatmann filter paper. To remove the diethyl ether remaining, the filtrate was put over rotator evaporator at 50 °C/30 min to get a pure NSE that was then collected in a sterile dark container and stored in refrigerator till use [29].

### 2.3. In Vitro Antimicrobial Susceptibility Assays

Disc diffusion, agar well diffusion and broth macrodilution were performed following Clinical and Laboratory Standards Institute (CLSI) guidelines for inoculum preparation, inoculation and data interpretation [30,31]. All assays were carried out in triplicate. Briefly, bacterial stocks were prepared by suspending 3–5 well-isolated overnight colonies from Baird-Parker agar plates into 3 mL of Mueller Hinton broth (MHB) (Oxoid, Basingstoke, Hampshire, England, UK) and the turbidity was adjusted to equal the 0.5 McFarland standard (10^8^ colony forming units, CFUs/mL). The adjusted suspensions were used as the final inocula for disc and agar well diffusion methods. For broth macrodilution assay, the suspensions were further diluted in MHB to be equivalent to 10^5^ CFU/mL.

Disc diffusion method was applied to evaluate the susceptibility of the recovered isolates against erythromycin (15 µg), doxycycline (30 µg), sulfamethoxazole/trimethoprim (1.25/23.75 µg), amoxicillin/clavulanic acid (20/10 µg), ciprofloxacin (5 µg), gentamicin (10 µg), rifampicin (5 µg), methicillin (5 µg), clindamycin (2 µg) and vancomycin (30 µg) and to preliminary determine the MRSA isolates using Mueller–Hinton agar (MHA, Oxoid, Basingstoke, Hampshire, England, UK) plates supplemented with 4% NaCl and ten commercially available antimicrobial discs (Oxoid, Basingstoke, Hampshire, England, UK). For the agar well diffusion assay used to determine the antimicrobial activity of NSE, MHA agar plates containing 4% NaCl were inoculated with the bacterial suspensions described above. Fifty microliters of NSE were further loaded into agar wells of six millimeters (mm) diameters punched in the culture medium. The plates for both methods were then incubated in an upright position at 37 °C for 24 h and the diameters of growth inhibition zones were measured in mm. Isolates those were resistant to at least three antimicrobial drugs from different classes were classified as MDR.

For the broth macrodilution test to estimate the minimal inhibitory concentration (MIC) of NSE, two-fold serial dilutions of NSE starting from 1024 μg/mL were prepared and then inoculated with the final inocula described above. The MICs of NSE were recorded as the lowest concentrations of the extract, where no visible growth of MRSA was observed.

### 2.4. Disc Diffusion Testing of MRSA Exposed to Sub-MICs of NSE

Bacterial sediments in broth macrodilution tube exposed to sub-MICs of NSE were harvested (NSE treated MRSA strains) and then the inoculum density was adjusted to the 0.5 McFarland turbidity standard. The respective treated strains were subjected to disc diffusion test using MHA plates and the previous antimicrobial discs. Results of inhibition zone diameters were recorded after incubating the plates at 37 °C for 24 h.

### 2.5. Synergism between Antibiotics and NSE

The combined effect of NSE with gentamicin, ciprofloxacin, doxycycline and erythromycin was evaluated by checkerboard method to obtain the fractional inhibitory concentration index (FICI) as detailed previously [22]. The FICI was interpreted as synergy, where it was ≤0.5 and antagonism, where it was >4. The FICI result between 0.5 and 1 was considered additive and a value between 1 and 4 was considered as indifferent or no effect [32]. All the experiments were repeated thrice.

### 2.6. In Vivo Assessment of NSE Efficacy

#### 2.6.1. Rabbits, Feeding and Experimental Design

200 weaned New Zealand male rabbits (32 days age) were commercially obtained from rabbit farms and randomly divided into 4 groups (5 replicates/group with 50 rabbits/group). The experimental groups included the control received the basal diet and other three groups fed dietary graded levels of NSE comprising 125, 250 and 500 mg/kg diets for 4 weeks. During the feeding period, the rabbits were reared in cages and fed pelleted diets with free access to feed and water. The diets were formulated following the rabbits nutrient recommendations [33]. The formulated experimental basal diet is shown in Table 1.

#### 2.6.2. Growth Performance

At the beginning of rearing period, initial body weight was recorded and then the body weight and feed intake of rabbits in each group were estimated weekly for calculating the growth performance traits of rabbits at the rearing period (60 days of age) including the feed intake (FI), body weight gain (BWG) and feed conversion ratio (FCR) as previously detailed [34,35,36,37].

#### 2.6.3. Sample Collection

At 60 days of age, blood samples were collected randomly from the ear veins of 15 rabbits per each group and used for sera separation via centrifugation at 2000 *g* for 10 min, then the collected sera were kept at −20 °C for sera bactericidal activity (SBA) assay. Moreover, experimental rabbits (n = 5/group) were sacrificed by cervical dislocation. Slaughtering and dissecting of rabbits were done as per the World Rabbit Science Association recommendations [38]. Slaughtered rabbits were skinned and eviscerated for collection of small intestinal contents for evaluating the activities of digestive enzymes. Moreover, jejunal and splenic tissues, which were kept in RNAlater^®^ (Sigma Aldrich, St. Louis, MO, USA) were used for further expression assays of tight junction protein (TJP) and immune related genes, respectively.

#### 2.6.4. Measurement of Digestive Enzymes and Serum Bactericidal Activities

Activities of chymotrypsin, α-amylase and lipase were estimated in the small intestinal contents via commercial kits (Nanjing Jiancheng Bioengineering Institute, Nanjing, China) following the producer’s manuals. Moreover, the collected sera from NSE fed rabbits were utilized for the measurement of SBA against MDR and MRSA strains as previously described [29]. Live bacterial cells were suspended in phosphate buffer saline (PBS) at a concentration of 3 × 10^2^ CFU/mL (stationary phase growth culture). Equal volumes of prepared bacterial cells in PBS and NSE supplemented rabbits’ sera or control rabbits’ sera were mixed and then incubated at 37 °C for 2 h with shaking. Consequently, the number of viable bacteria was calculated, in duplicate, by counting the colonies from the resultant mixture (10 µL) on Baird-Parker agar plates after incubation at 37 °C for 48 h. The bactericidal activity of sera from NSE supplemented rabbits was expressed as percentages of CFUs in test groups to that in the non-supplemented control group. The colony counts were used to calculate the sera bactericidal index (SBI). Sera index was defined as the dilution of the serum that resulted in half as many colonies as are seen with the control.

#### 2.6.5. Reverse Transcription Quantitative Real-Time PCR (qRT-PCR) Assays

RNA was extracted from jejunal and splenic tissues via QIAamp RNeasy Mini kit (Qiagen, Hilden, Germany) following the manufacturer’s procedures. The quantity and purity of RNA were evaluated by Nano Drop 2000 spectrophotometer (Thermo Fisher Scientific Inc., Waltham, MA, USA). The expression levels of genes encoding TJP including occludin, junctional adhesion molecule-2 (JAM-2), claudins-1 (CLDN-1) and mucin-2 (MUC-2), cytokines comprising interleukin-6 (IL-6), IL-1β, IL-8, IL-10 and tumor necrosis factor-alpha (TNF-α), toll like receptor (TLR) 2 and 4 and defensin beta 1 (DEFB1) were assessed, in triplicate, via qRT-PCR assay using 2x QuantiTect SYBR Green RT-PCR Kit (Qiagen, Hilden, Germany). All qRT-PCR reactions were done on Stratagene Mx3005P real-time thermal cycler ((Stratagene, La Jolla, CA, USA). Transcript levels of the investigated genes were normalized to the housekeeping gene, glyceraldehyde 3-phosphate dehydrogenase (*GAPDH*) expression. The sequences of the primers of target genes are listed in Table 2. After qRT-PCR reactions, the specificity of PCR amplifications was confirmed by melting curves analyses. The relative changes in the levels of gene expression were assessed using the 2^−ΔΔCt^ method [39].

#### 2.6.6. Challenge Trial

Before the challenge trial, all rabbits were ensured to be free from MRSA. At the end of feeding period (60 d of age), experimental rabbits (15 rabbits per group) were subcutaneously challenged with MDR and MRSA fresh inocula containing an average of 5 × 10^6^ CFUs two days apart. The challenged rabbits were kept under observation for a total of 2 weeks for development of any clinical symptoms and the mortality rates were then calculated. Re-isolation and identification of the challenging strain were carried out for establishment of the experimental infection. At the end of the observation period, all rabbits were euthanized and CFUs of MRSA strains were determined in any developed abscesses and per gram of liver, spleen and heart tissues after culturing onto Baird Parker medium.

### 2.7. Statistical Analysis

General linear model of SPSS was used for exploring our data and Tukey’s post-hoc test was used to assess the statistical significance variations among the treatment groups. Homogeneity and normality among tested groups were estimated via Levene’s and Shapiro–Wilk’s tests, respectively. The achieved data was defined as standard error of means (SEM) and the significance test results were detected at *p* < 0.05.

## 3. Results

### 3.1. Characterization and Incidence of MRSA Isolates

Based on standard conventional bacteriological methods, 23 *S. aureus* isolates were identified from 67 investigated samples with an overall prevalence rate of 34.3%. Of note, a high prevalence rate of *S. aureus* isolates (60%) was observed in animal origin samples. Moreover, *S. aureus* isolation rate from the 52 specimens collected from human origin was found to be 26.9%. All the recovered isolates exhibiting yellow colonies on Mannitol Salt Agar and black colonies on Baird-Parker agar were β-hemolytic on blood agar, formed characteristic golden yellow pigments on milk agar, Gram-positive cocci arranged in grape-like clusters and catalase and tube coagulase tests positive. Final molecular confirmation of the investigated isolates was applied via PCR amplification of *nuc* and *mecA* genes and the results revealed that all our examined isolates were confirmed to be MRSA. The rate of MRSA isolation with regard to the animal samples was the highest in meat products (66.7%). Furthermore, the high proportion of MRSA isolates from human source was from cerebrospinal fluid samples (50%)

### 3.2. In Vitro Antimicrobial Susceptibility Patterns of MRSA Isolates

In the disk diffusion test using methicillin disk, all the recovered isolates were considered to be MRSA. Antimicrobial susceptibility results reveled that MRSA resistance was noticed most commonly to amoxicillin/clavulanic acid and sulfamethoxazole/trimethoprim with percentages of 47.8 and 39.1%, respectively; meanwhile, a lower resistance rate of MRSA isolates was observed against gentamicin (4.3%). Fortunately, and from the therapeutic perspective, resistance to vancomycin was not detected among our MRSA isolates. The MDR pattern was observed in most human (57.1%) and animal (55.6%) MRSA isolates with an overall prevalence rate of 56.5% (Supplementary Table S1).

### 3.3. Antimicrobial Activity of NSE against MRSA Isolates

The susceptibility of MRSA strains isolated from animal samples and human subjects to NSE is depicted in Table 3 and Table 4. Seven out of nine MRSA isolates of animal origin (77.8%) were susceptible to NSE with recorded inhibition zones’ diameters ranging from 15 to 28 mm as estimated by agar well diffusion method and MIC values varying from 8 to 1024 ug/mL as detected by broth macrodilution test. Only two MRSA isolates from cows’ milk samples were resistant to NSE and they were inhibited by most of the tested antimicrobials (Table 3). Regarding the susceptibility of MRSA strains isolated from human origin to NSE, 9 out of 14 (64.3%) were sensitive with inhibition zones’ diameters ranging from 15 to 24 mm and corresponding MIC values ranged between 16 to 1024 ug/mL. Five MRSA isolates from pus and urine samples were sensitive to NSE; four of them were MDR (Table 4).

### 3.4. Antimicrobial Susceptibility Profiles of MRSA after Exposure to Sub-MICs of NSE

Comparison of antibiogram of MRSA isolates exposed to sub-MICs of NSE with that of the original isolates, which were not exposed to NSE revealed that MRSA strains that resisted 1–7 antimicrobials became highly sensitive to the antimicrobials to which they were resistant after treatment with NSE sub-MICs.

### 3.5. Synergism Assays between NSE and Antibiotics

The results of the checkerboard method demonstrated that combination of NSE with antibiotics exhibited synergistic interactions against MRSA strains with FICI ≤ 0.5. Among the screened antibiotics, ciprofloxacin or gentamicin and ciprofloxacin, doxycycline or gentamicin showed distinct synergistic effects in combination with NSE against MRSA strains from pus and urine samples, respectively.

### 3.6. Growth Performance and Digestive Enzymes’ Activities

The results of the growth performance parameters of rabbits are shown in Table 5. At the end of growing period (60 d), the prominent significant (*p* < 0.05) increase in BWG was observed in rabbits received diets supplemented with NSE at the levels of 250 and 500 mg/kg. Meanwhile, there was no significant difference between the BWG of rabbits fed 125 mg/kg of NSE and those fed control diet. Moreover, rabbits fed diets supplemented with NSE at the level of 500 mg/kg exhibited the most improved (*p* < 0.05) FCR. The data regarding analysis of digestive enzymes’ activities are illustrated in Table 5. The results indicated that amylase and lipase activities were significantly (*p* < 0.05) increased post NSE supplementation in a dose dependent manner. Moreover, chymotrypsin activity reached its peak in rabbits received 500 mg/kg of NSE.

### 3.7. Bactericidal Activity of Sera of NSE Fed Rabbits against MRSA Isolates

The bactericidal activity of sera of rabbits fed NSE supplemented diets examined against 5 representative MDR and MRSA isolates from different sources is clarified in Table 6. Sera bactericidal activity increased to 86.1% in NSE fed rabbits compared with 43% in the non-supplemented control rabbits. Serum bactericidal indices (SBIs) against three MRSA isolates from sputum, pus and sausage samples (code No. 1 Sp, 1 Pu and 5 Sg) those were sensitive to NSE ranged from 31.06–43.2. Meanwhile, SBIs against the other two MRSA isolates from cow milk and pus samples (code No. 4 M and 3 Pu) those were resistant to NSE were 1.2 and 0.7, respectively. After culturing on Baird-Parker agar plates, the colonies of MRSA isolates those were exposed to sera from rabbits fed NSE supplemented diets were smaller in size, fewer in number and of rough texture compared to those of MRSA isolates exposed to sera of non-supplemented control rabbits, which were larger in size, higher in number and of smooth surface.

### 3.8. Gene Expression Analysis

**Figure 1:**
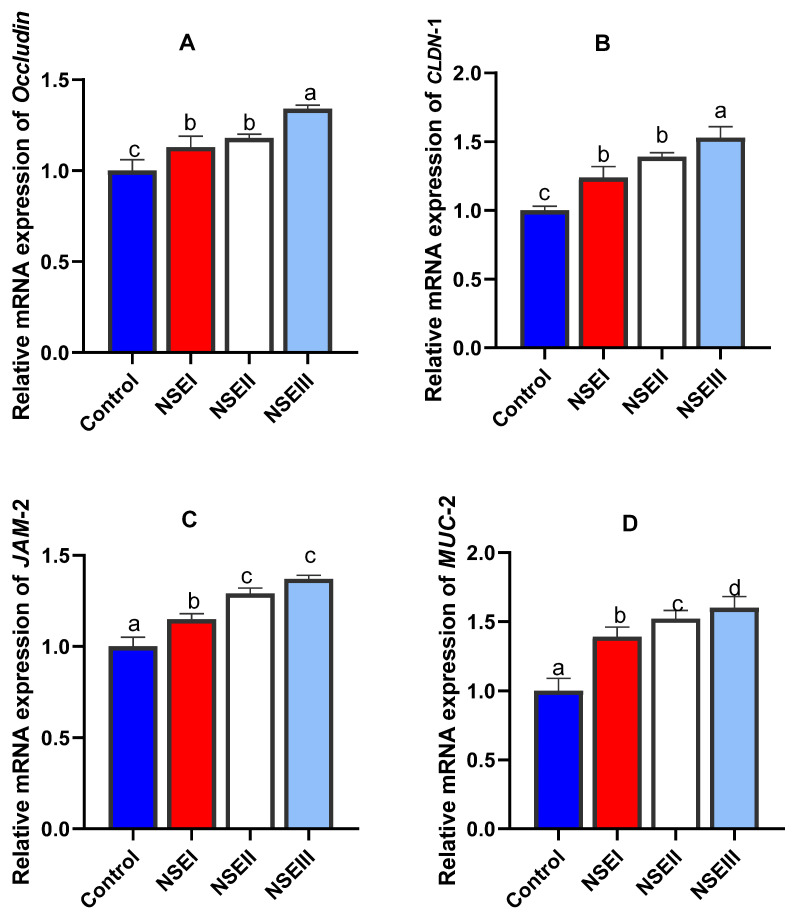
Gene expression of occludin (**A**), claudins-1 (CLDN-1, (**B**)), junctional adhesion molecule-2 (JAM-2, (**C**)) and mucin-2 (MUC-2, (**D**)) quantified using qRT-PCR in the jejunal tissues of rabbits fed various levels of *N. sativa* oil extract (NSE) at 60 d of age. Values represent means with their SE in bars. Control: rabbits offered the basal diet, NSEI, NSEII and NSEIII: rabbits fed graded levels of NSE comprising 125, 250 and 500 mg/kg diets, respectively. a–d Means carrying different superscript letters indicate statistical significance (*p* < 0.05).

Concerning the mRNA expression of intestinal barrier related genes, our data revealed that the maximum (*p* < 0.05) transcriptional levels of occludin (Figure 1A) and *CLDN-1* (Figure 1B) genes were noticed in rabbits received 500 mg/kg of NSE. Moreover, rabbits fed 250 and 500 mg/kg of NSE had higher significant (*p* < 0.05) expression levels of *JAM-2* unlike those fed control diet (Figure 1C). Supplementation of rabbits with NSE had a dose dependent effect on the *MUC-2* expression level (Figure 1D).

**Figure 2:**
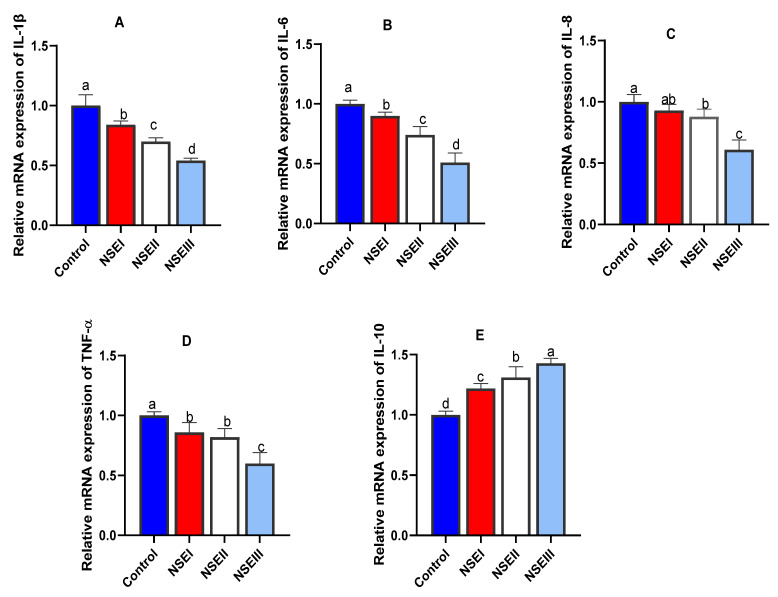
Relative mRNA expression levels of interleukin-1β IL-1β, (**A**), IL-6 (**B**), IL-8 (**C**), tumor necrosis factor-alpha TNF-α, (**D**) and IL-10 (**E**) detectable by qRT-PCR in the splenic tissues of rabbits fed various levels of *N. sativa* oil extract (NSE) at 60 d of age. Values represent means with their SE in bars. Control: rabbits offered the basal diet, NSEI, NSEII and NSEIII: rabbits fed graded levels of NSE comprising 125, 250 and 500 mg/kg diets, respectively. a–d Means carrying different superscript letters indicate statistical significance (*p* < 0.05).

The findings of cytokines gene expression analysis are shown in Figure 2. Notably, the relative expression levels of *IL-1β* (Figure 2A) and *IL-6* (Figure 2B) genes were significantly (*p* < 0.05) downregulated with increasing dietary levels of NSE. Furthermore, rabbits fed NSE at the level of 500 mg/kg displayed the most prominent (*p* < 0.05) decrease in the expression levels of *IL-8* (Figure 2C) and *TNF-α* (Figure 2D) genes. Meanwhile, supplementing rabbits with NSE dose dependently upregulated (*p* < 0.05) the transcriptional level of *IL-10* gene (Figure 2E).

**Figure 3:**
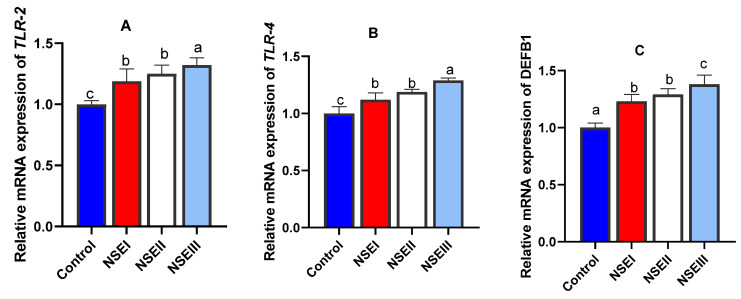
Reverse transcription quantitative real-time PCR analysis of toll like receptor (TLR)-2 (**A**), TLR-4 (**B**) and defensin beta 1 (DEFB1, (**C**)) mRNA expression in the spleen of rabbits fed various levels of *N. sativa* oil extract (NSE) at 60 d of age. Values represent means with their SE in bars. Control: rabbits offered the basal diet, NSEI, NSEII and NSEIII: rabbits fed graded levels of NSE comprising 125, 250 and 500 mg/kg diets, respectively. a–c Means carrying different superscript letters indicate statistical significance (*p* < 0.05).

The mRNA expression analysis of *TLR2* (Figure 3A) and *TLR4* (Figure 3B) related genes were significantly (*p* < 0.05) increased especially with higher NSE supplementation levels. Moreover, the maximum significant (*p* < 0.05) expression level of *DEFB1* gene (Figure 3C) was detected in rabbits fed 500 mg/kg of NSE.

### 3.9. Clearance of Rabbits’ Internal Organs from MRSA Infection by NSE

After 2 weeks observation period of MRSA challenged and non-challenged rabbits, no detectable clinical signs or skin abscess were seen in rabbits fed NSE supplemented compared to subcutaneous and skin abscesses and reduction in rabbits’ activity in control rabbits. No CFUs were detected in liver, spleen and heart tissues of NSE fed rabbits after culturing on Baird-Parker agar media. Meanwhile, the average CFUs per gram of the liver tissues of the control rabbits was calculated to be 1.07 × 10^5^.

## 4. Discussion

Recently, numerous prospective approaches had been encouraged to avoid the weaning associated stressful contentions in rabbits. Moreover, the side effects and developing resistance restrict the usage of antibiotics and direct efforts to purify and assay alternative active antibacterial components from natural herbs [40,41,42]. Phytobiotics are supposing a position of crucial importance in rabbits. Among them, *N. sativa* is commonly named a miracle herb, because its seed or even its extract has many bioactive ingredients. Previously, *N. sativa* had been found to have therapeutic potentials and promising antibacterial effects against *S. aureus* [43]. However, NSE were not fully investigated against MDR-MRSA strains as recommended previously [44]. Moreover, there are scarce data considering the promising immunostimulant role of NSE and to which extent it can augment rabbits’ growth and modify molecular aspects of digestive, tight junction and cytokines related genes to fight against MRSA experimental infection. Herein, the antimicrobial activity of NSE against MRSA strains from both human clinical samples and animal food-products were tested. NSE showed promising antimicrobial activity as it was able to inhibit most of the strains isolated from human samples or animals’ food-products. In addition, both gentamicin and NSE were the most effective among the tested antimicrobials in the synergism study and it could be recommended for administration in combination with NSE to treat MRSA infected cases. Previously, a synergism was found to be effective when ampicillin, cephalexin, chloramphenicol, and tetracycline was combined with TQ and THQ [45]. Herein, MRSA strains were sensitive to NSE and they were inhibited with zones ranging from 15–28 mm and MIC values from 8 to 1024 ug/mL. Previously, *N. sativa* methanol extracts produced an inhibition zone of 19 mm at a concentration 100 mg/mL with no activity at lower concentrations [20] indicating that the antimicrobial activity of the extract is dose-dependent. This difference in inhibition zones and MIC values may be attributed to strains origin, the used extraction method or the source of the seeds.

Interestingly, MRSA became more sensitive to all the tested antimicrobials after exposure to sub-MICs of NSE confirming the necessity of synergism between antimicrobials and natural herbal products. Potentiating the antimicrobials efficacy against strains under effects of sub-MIs of the extract suggests that it has significant impact on the envelops of bacterial cell [43] or may induce molecular changes in its structural components. Cell wall morphological changes and plasma membrane disruption were detected previously by transmission electron microscopy after exposure of molds to different concentrations of NSE [45].

On the topic of the *in vivo* efficacy of NSE on rabbits’ growth performance, our results demonstrated its positive role on their BWG and FCR at 60 days of age. Similarly, enhancement of FCR was achieved after supplementing 4 g/kg of black cumin seed in four weeks old broilers [46]. Furthermore, a stimulating effect of black seed on digestive system with a consequence of superior absorption and performance of broiler chickens were previously proved [47]. Additionally, enhancing the activities of digestive enzymes came in parallel with previous growth promoting results [48]. In agreement with our findings, dietary supplementation of *N. sativa* increased the bile flow rate resulting in activation of pancreatic lipases and assisting in digestion and absorption of fat [49]. Similarly, Hannan et al. described the stimulating role of *N. sativa* bioactive compounds on digestion [50]. This could be attributed to its role as a strong stimulant on digestive enzymes [51] and its potency in increasing the retention of nutrients with a positive reflection on their utilization. Moreover, the promising effects of *N. sativa* on growth performance are assumed to be due to its high nutritive value along with its pharmacologically active compounds [51].

The tight junctions represent the key components of the gut barrier function as they act as functional and physical barriers against the entrance of pathogenic bacterial species and other toxic molecules [52]. The intestinal barrier aspects are reflected by various markers including tight junction molecules, mucin and defensins. Additionally, TLR signaling boosts the tight junction integrity, which is mediated by accelerated expression of key tight junction related genes. In the current study, the mRNA expression analysis of selected intestinal barrier function-related genes demonstrated that NSE supplementation increased the transcriptional levels of occludin, *CLDN-1*, *JAM-2*, *MUC-2* and defensin genes. Moreover, the mRNA expression analysis of *TLR2* and *TLR4* related genes were increased post higher NSE inclusion. Interestingly, the expression results of all tight junction molecules measured are closely correlated. Similarly, recent studies displayed the intestinal barriers protective efficacy of plant extracts [11,12,53] owing to their effects on sustaining the integrity of junction complexes. Focusing on the subject of interest, bioactive compounds of *N. sativa* are capable for improving intestinal barrier functions [54]. The herbal extracts could argument the intestinal barrier function and the nutrient transportation in poultry via numerous molecular mechanisms of actions those might regulate genes expression of CLDN-1, JAM-2 and occludin [55].

Interactive impacts of dietary plant derived bioactive compounds on superior immune responsiveness through modulating the expression of several cytokines, pattern recognition receptors and mucin can influence the maintenance of intestinal integrity and barrier functions [56]. Notably, *N. sativa* exerts immunostimulatory effects in various inflammatory and immunologic diseases in animals [57] and its key constituent, thymoquinone, significantly influences the proinflammatory cytokines such as IL-6, IL-8 and TNF-α and anti-inflammatory IL-10 preventing the intestinal inflammatory changes [54]. Our results exhibited that the transcriptional levels of *IL-1b*, *IL-6*, *IL-8* and *TNF-α* were significantly lower and that of *IL-10* was higher post dietary NSE supplementation in rabbits. In accordance, thymoquinone can regulate the inflammatory cells trafficking through modifying the expression of chemokines and/or cytokines with a suppressing consequences on the inflammatory immune response [57]. Moreover, boosted immune reactions in broiler chickens were achieved after dietary inclusion of black cumin (*Nigella sativa* L.) powder [58]. In a comparable study, feeding of rabbits on diets containing black cumin seed greatly modulated their immune responses [59]. Similarly, dietary supplementation with *N. sativa* in male Wistar rats mediated immunological function [60]. These observations might explain the anti-inflammatory activity of black cumin [54]. Previously, the positive impacts of *N. sativa* on the immune system were achieved by improving the antibody response and restoring the inflammation and immunological attacks [61].

Previous findings describing that *N. sativa* bioactive ingredients can be detected in serum shortly after feeding [62,63] supported our SBA post NSE supplementation (74.2 to 86.1% with NSE compared with 34% in the control). Detection of *N. sativa* in rabbits’ sera shortly after feeding ensures the produced bacteriolysis and tissue clearance of infection as indicated in bacterial cells morphology and colony characters [64]. In the current study, NSE protected challenged rabbits and prevented lesions and MRSA colonization in their skin and organs with detectable improvement in general health condition. Concerning the animal protection against MRSA induced experimental infection after NSE supplementation, we have previously reported that it was able to reduce the survival of bacteria, increase nitric oxide production and enhance phagocytic capacity of NSE-exposed macrophages culture [65]. This modulation in innate immune response function may be a possible cause of the protection line against the MRSA induced infection. Previously, *N. sativa* was shown to have immunomostimulatory activities that might play effective roles in controlling MRSA post challenge. These activities include (i) enhancement of cytokines production such as interferon and interleukin-1β, which have been known to be macrophage activators and to stimulate immune cells microbicidal activity [66], (ii) helping in lysosome binding to phagosome and in phagolysosome formation [57], or (iii) initiation of cell signaling that results in microbicidal mediators production such as toxic oxygen radicals or nitric oxide intermediates [65,67,68].

## 5. Conclusions

The findings of our study concluded that NSE had an *in vitro* potential promising antibacterial activity against MRSA either alone or in synergism with antibiotics. Herein, the favorable actions of NSE on growth performance of rabbits proposed to be throughout its upregulating role on digestive enzymes’ genes expression. Furthermore, our outcomes supported the immune enhancement of NSE via controlling the expression of regulators that controlled the progress of inflammation and augmenting those maintained the intestinal barrier function. Sera from NSE-fed rabbits had bactericidal activities that were able to fight against MRSA challenge and effectively protect rabbits’ internal organs from bacterial colonization and lesions.

## Figures and Tables

**Table 1:** Feed ingredients and nutrition levels of the control experimental diet.

Ingredient	%
Yellow corn	9.50
Barley grains	18.60
Soybean meal, 44%	16.50
Berseem hay	34.00
Wheat bran	15.80
Molasses	2.80
Premix *	0.3
Calcium dibasic phosphate	1.5
Common salt	0.5
Antitoxin	0.3
Anticoccidial	0.2
Nutrient levels	
Digestable energy, Kcal/Kg	2587
Crude protien	16.55
Ether extract	2.26
Crude fiber	12.60
Ca	1.02
Phosphorus	0.62
Lysin	0.76
Methionine	0.25

* Premix: one kg consists of vitamins; D3: 8000 IU, E: 3300 mg, A: 27,000 IU, E: 30.5 IU, B1: 230 mg, B2: 200 mg, B12: 25 mg and B6: 10 mg, calcium: 250 mg, nicotinic acid: 35,000 mg, choline: 2000 mg, magnesium: 20 g, Cu: 800 mg, Mn: 2000 mg, I: 60 mg, Co: 5 mg, Fe: 2000 mg, Se: 5 mg and Zn: 6000 mg.

**Table 2:** Primer sequences utilized for qRT-PCR analysis.

Specificity/Encoding Gene	Primer Sequence (5′-3′)	Accession No
Tight junction proteins		
*MUC-2*	F: TATACCGCAAGCAGCCAGGT R: GCAAGCAGGACACAGACCAG	L41544.1
*JAM-2*	F: ATATCGCAGGTGTCCTGGAA R: GAGCATAGCACACGCCAAG	XM_017346699
*CLDN-1*	F: GGAGCAAAAGATGCGGATGG R: AATTGACAGGGGTCAAAGGGT	NM_001089316.1
*Occludin*	F: GCAAGAGGCCGTATCCAGAG R: AGTCCGTCTCGTAGTGGTCT	XM_008262320.1
Cytokines		
*IL-6*	F: GCCAACCCTACAACAAGA R: AGAGCCACAACGACTGAC	NC_013678
*IL-8*	F: CTCTCTTGGCAACCTTCCTG R: TTGCACAGTGAGGTCCACTC	KT216053.1
*IL-10*	F: AAAAGCTAAAAGCCCCAGGA R: CGGGAGCTGAGGTATCAGAG	NM001082045.1
*IL-1β*	F: TTCCGGATGTATCTCGAGCA R: GTGGATCGTGGTCGTCTTCA	NC_013670
*TNF-α*	F: CTGCACTTCAGGGTGATCG R: CTACGTGGGCTAGAGGCTTG	XM_008262537.2
*TLR-2*	F: TGCCTCCTTGTTACCTATGC R: AGATGAAGTTGTTCCCTCCG	NM_00108271
*TLR-4*	F: AGATGAAGTTGTTCCCTCCG R: GTGGGCTTAGAACAACTGGAAC	NM_001082732.2
*DEFB1*	F: AGCCTGTCTGCCTGGAGTAG R: GATGAGGAGAGGCTTCATGG	XM017337690.1
House keeping		
*GAPDH*	F: TGTTTGTGATGGGCGTGAA R: CCTCCACAATGCCGAAGT	NC_013676.1

*CLDN-1:* claudins-1, *MUC-2*: mucin-2, *JAM-2*: junctional adhesion molecule- 2, *IL*: interleukin, *TNF-α:* tumor necrosis factor-alpha, *TLR*: toll like receptor, *DEFB1*: defensin beta1, *GAPDH*: glyceraldehyde 3-phosphate dehydrogenase.

**Table 3:** Susceptibility of resistant MRSA isolates from cows’ milk and meat products to NSE.

Sample Source	MRSA Tested Isolates	Antimicrobial Resistance Profile	NSE Antimicrobial Activity	
			Zone Diameter (mm)	MIC (ug/mL)
Cows’ milk	1 M	ME, E, DO, DA, AMC	28	16
	2 M	ME, AMC	18	128
	3 M	ME, AMC	30	8
	4 M	ME, SXT, CIP	- (R)	1024
	5 M	ME	12 (R)	512
Meat products	1 Mm	ME, AMC, CIP	18	128
	2 Br	ME, DO, CIP	18	128
	3 Br	ME, AMC, DO, RF	18	128
	5 Sg	ME, RF, E, DA, AMC	15	256

MRSA: methicillin-resistant *Staphylococcus aureus*, M: milk, Mm: minced meat, Br: burger, Sg: sausage, ME: methicillin, E: erythromycin, DO: doxycycline, DA: clindamycin, AMC: amoxicillin/clavulanic acid, SXT: sulfamethoxazole/trimethoprim, CIP: ciprofloxacin, RF: rifampicin, NSE: *Nigella sativa* extract, R: resistant, MIC: minimum inhibitory concentration.

**Table 4:** Antibacterial activity of NSE against resistant MRSA isolates from human clinical samples.

Sample Source	MRSA Tested Isolates	Antimicrobial Resistance Profile	NSE Antimicrobial Activity	
			Zone Diameter (mm)	MIC (ug/mL)
Pus	1 Pu	ME, RF, DO, CN, SXT, AMC, E	15	256
	2 Pu	ME, SXT, AMC, DA, RF	15	256
	3 Pu	ME, DO, SXT, DA, CIP	- (R)	1024
	4 Pu	ME	12 (R)	512
	5 Pu	ME	20	64
Urine	1 U	ME, E, RF, AMC, SXT	12 (R)	512
	2 U	ME, E, SXT	12 (R)	512
	3 U	ME, E, SXT, CIP	8 (R)	1024
Sputum	1 Sp	ME, E, DO, SXT, AMC, DA, CIP	24	16
	2 Sp	ME, CIP	17	128
Diabetic foot	1 Df	ME, DO	20	64
Burn swab	1 Bs	ME, E, DO, SXT, AMC, DA, CIP	16	256
Blood	1 Bl	ME	16	256
Cerebrospinal fluid	1 CSF	ME	18	128

MRSA: methicillin-resistant *Staphylococcus aureus*, Pu: pus, U: urine, Sp: sputum, Df: diabetic foot, Bs: burn swab, Bl: blood, CSF: cerebrospinal fluid, ME: methicillin, E: erythromycin, DO: doxycycline, DA: clindamycin, AMC: amoxicillin/clavulanic acid, SXT: sulfamethoxazole/trimethoprim, CIP: ciprofloxacin, RF: rifampicin, CN: gentamicin, NSE: *Nigella sativa* extract, R: resistant, MIC: minimum inhibitory concentration.

**Table 5:** Effects of various levels of NSE on rabbits’ growth performance aspects and digestive enzymes’ activities.

Parameter	Experimental Group				*p* Value	SEM
	Control	NSEI	NSEII	NSEIII		
Initial body weight	804	808	766	806	0.06	10.29
Growing period (30–60 d)						
Body weight, g	2036 ^c^	2060 ^c^	2237 ^b^	2346 ^a^	<0.03	15.14
BWG, g	1232 ^c^	1251 ^c^	1471 ^b^	1537 ^a^	<0.04	18.16
FI, g	3033 ^b^	3030 ^b^	3372 ^a^	3351 ^a^	0.04	20.83
FCR	2.46 ^a^	2.42 ^a^	2.30 ^b^	2.18 ^c^	0.001	<0.001
Digestive enzymes						
Chymotrypsin (U/gprot)	69.96 ^c^	79.99 ^b^	83.69 ^ab^	85.36 ^a^	0.03	0.15
Amylase (U/gprot)	1.95 ^c^	2.10 ^c^	2.56 ^b^	2.96 ^a^	0.001	0.30
Lipase (U/gprot)	39.64 ^d^	42.55 ^c^	47.6 ^b^	49.67 ^a^	0.04	0.22

BWG: body weight gain, FI: feed intake, FCR: feed conversion ratio, control: rabbits offered the basal diet, NSEI, NSEII and NSEIII: rabbits fed graded levels of NSE comprising 125, 250 and 500 mg/kg diets, respectively. ^a–d^ Means in a row carrying different superscript letters indicate statistical significance (*p* < 0.05).

**Table 6:** Bactericidal activity of sera of NSE fed rabbits against MRSA isolates.

Sample Source	MRSA Code No.	Serum Bactericidal Capacity (%)		Bactericidal Index
		Control	NSE Fed Rabbits	
Sputum	1 Sp	43	86.1	43.2
Pus	1 Pu		81.0	32.0
Sausage	5 Sg		74.2	31.1
Cow milk	4 M		55.0	1.2
Pus	3 Pu		50.0	0.7

MRSA: methicillin-resistant *Staphylococcus aureus*, Sp: sputum, Pu: pus, Sg: sausage, M: milk, NSE: *Nigella sativa* extract.

## Data Availability

The data presented in this study are available on request from the corresponding author.

## Supplementary Materials

The following supporting information can be downloaded at: https://www.mdpi.com/article/10.3390/ani12192635/s1, Table S1: Antimicrobial resistance patterns of MRSA isolates from animal and human origins.

## References

[1] Christaki E., Marcou M., Tofarides A. (2020). Antimicrobial resistance in bacteria: Mechanisms, evolution, and persistence. J. Mol. Evol..

[2] Girard M., Bee G. (2020). Invited review: Tannins as a potential alternative to antibiotics to prevent coliform diarrhea in weaned pigs. Animal.

[3] Alandiyjany M.N., Abdelaziz A.S., Abdelfattah-Hassan A., Hegazy W.A., Hassan A.A., Elazab S.T., Mohamed E.A., El-Shetry E.S., Saleh A.A., ElSawy N.A. (2022). Novel *In vivo* assessment of antimicrobial efficacy of ciprofloxacin loaded lesoporous silica nanoparticles against *Salmonella* Typhimurium infection. Pharmaceuticals.

[4] Ibrahim D., Eldemery F., Metwally A.S., Abd-Allah E.M., Mohamed D.T., Ismail T.A., Hamed T.A., Al Sadik G.M., Neamat-Allah A.N., Abd El-Hamid M.I. (2022). Dietary eugenol nanoemulsion potentiated performance of broiler chickens: Orchestration of digestive enzymes, intestinal barrier functions and cytokines related gene expression with a consequence of attenuating the severity of *E. coli* O78 infection. Front. Vet. Sci..

[5] Chambers H.F., DeLeo F.R. (2009). Waves of resistance: Staphylococcus aureus in the antibiotic era. Nat. Rev. Microbiol..

[6] Van Hal S.J., Jensen S.O., Vaska V.L., Espedido B.A., Paterson D.L., Gosbell I.B. (2012). Predictors of mortality in *Staphylococcus aureus* bacteremia. Clin. Microbiol. Rev..

[7] Gould I.M., David M.Z., Esposito S., Garau J., Lina G., Mazzei T., Peters G. (2012). New insights into meticillin-resistant *Staphylococcus aureus* (MRSA) pathogenesis, treatment and resistance. Int. J. Antimicrob. Agents.

[8] Ammar A., El-Hamid M., Eid S.E., Oksh A.S.E. (2015). Insight into antimicrobial resistance and virulence genes of emergent multidrug resistant avian pathogenic *Escherichia coli* in Egypt: How closely related are they. Rev. Med. Vet..

[9] Ammar A.M., El-Naenaeey E.-S.Y., El-Hamid M.I.A., El-Gedawy A.A., Elmalt R.M. (2021). Campylobacter as a major foodborne pathogen: A review of its characteristics, pathogenesis, antimicrobial resistance and control. J. Microbiol. Biotechnol. Food Sci..

[10] Hashem Y.M., El-Hamid M.I.A., Awad N.F., Ibrahim D., Elshater N.S., El-Malt R.M., Hassan W.H., Abo-Shama U.H., Nassan M.A., El-Bahy S.M. (2022). Insights into growth-promoting, anti-inflammatory, immunostimulant, and antibacterial activities of Toldin CRD as a novel phytobiotic in broiler chickens experimentally infected with *Mycoplasma gallisepticum*. Poult. Sci..

[11] El-Hamid M.I.A., Ibrahim D., Hamed R.I., Nossieur H.H., Elbanna M.H., Baz H., Abd-Allah E.M., Oksh A.S.E., Ibrahim G.A., Khalifa E. (2022). Modulatory impacts of multi-strain probiotics on rabbits’ growth, nutrient transporters, tight junctions and immune system to fight against *Listeria monocytogenes* infection. Animals.

[12] Khater S.I., Lotfy M.M., Alandiyjany M.N., Alqahtani L.S., Zaglool A.W., Althobaiti F., Ismail T.A., Soliman M.M., Saad S., Ibrahim D. (2022). Therapeutic potential of quercetin loaded nanoparticles: Novel insights in alleviating colitis in an experimental DSS induced colitis model. Biomedicines.

[13] Aljazzar A., El-Hamid M.I.A., El-Malt R.M., El-Gharreb W.R., Abdel-Raheem S.M., Ibrahim A.M., Abdelaziz A.M., Ibrahim D. (2022). Prevalence and antimicrobial susceptibility of *Campylobacter* species with particular focus on the growth promoting, immunostimulant and anti-*Campylobacter jejuni* activities of eugenol and trans-cinnamaldehyde mixture in broiler chickens. Animals.

[14] Ibrahim D., Moustafa A., Metwally A.S., Nassan M.A., Abdallah K., Eldemery F., Tufarelli V., Laudadio V., Kishawy A.T. (2021). Potential application of cornelian cherry extract on broiler chickens: Growth, expression of antioxidant biomarker and glucose transport genes, and oxidative stability of frozen meat. Animals.

[15] Ammar A., Attia A., El-Hamid M.A., El-Shorbagy I., El-Kader S.A. (2016). Genetic basis of resistance waves among methicillin resistant Staphylococcus aureus isolates recovered from milk and meat products in Egypt. Cell. Mol. Biol..

[16] Toama M.A., El-Alfy T.S., El-Fatatry H.M. (1974). Antimicrobial activity of the volatile oil of *Nigella sativa* Linneaus seeds. Antimicrob. Agents Chemother..

[17] Kumar T.S., Negi P., Sankar K.U. (2010). Antibacterial activity of “*Nigella sativa* L.” seed extracts. Br. J. Pharmacol. Toxicol..

[18] Chaieb K., Kouidhi B., Jrah H., Mahdouani K., Bakhrouf A. (2011). Antibacterial activity of thymoquinone, an active principle of *Nigella sativa* and its potency to prevent bacterial biofilm formation. BMC Complement. Altern. Med..

[19] Abu-Al-Basalc M.A. (2009). In vitro and iin vivo anti-microbial effects of *Nigella sativa* Linn. seed extracts against clinical isolates from skin wound infections. Am. J. Appl. Sci..

[20] Sharikh S.M., Rao J.U.P., Ackshaya M.S. (2020). A study of antibacterial effect of *Nigella Sativa* seed extract on clinical isolates of methicillin-resistant *Staphylococcus aureus* (MRSA). Indian J. Public Health Res. Dev..

[21] Nagi A., Mariana N., Hana F., Rasedee A. (2008). Extract of essential iol from *Nigella sativa* using superfacial carbon dioxide: Study of antibacterial activity. Am. J. Pharmacol. Toxicol..

[22] El-Hamid M.I.A., El-Sayed M., Ali A.R., Abdallah H., Arnaout M.I., El-Mowalid G.A. (2019). Marjoram extract down-regulates the expression of *Pasteurella multocida* adhesion, colonization and toxin genes: A potential mechanism for its antimicrobial activity. Comp. Immunol. Microbiol. Infect. Dis..

[23] Piras A., Rosa A., Marongiu B., Porcedda S., Falconieri D., Dessi M.A., Ozcelik B., Koca U. (2013). Chemical composition and iin vitro bioactivity of the volatile and fixed oils of *Nigella sativa* L. extracted by supercritical carbon dioxide. Ind. Crops Prod..

[24] Halawani E. (2009). Antibacterial activity of thymoquinone and thymohydroquinone of *Nigella sativa* L. and their interaction with some antibiotics. Adv. Biol. Res..

[25] El-Hamid M.A., Bendary M., Merwad A., Elsohaby I., Mohammad Ghaith D., Alshareef W. (2019). What is behind phylogenetic analysis of hospital-, community-and livestock-associated methicillin-resistant *Staphylococcus aureus*?. Transbound. Emerg. Dis..

[26] El-Hamid A., Marwa I., Sewid A.H., Samir M., Hegazy W.A., Bahnass M.M., Mosbah R.A., Ghaith D.M., Khalifa E., Ramadan H. (2022). Clonal diversity and epidemiological characteristics of ST239-MRSA Strains. Front. Cell. Infect. Microbiol..

[27] El-Hamid M.I.A., Bendary M. (2015). Comparative phenotypic and genotypic discrimination of methicillin resistant and susceptible *Staphylococcus aureus* in Egypt. Cell. Mol. Biol..

[28] El-Aziz N.K.A., El-Hamid M.I.A., Bendary M.M., El-Azazy A.A., Ammar A.M. (2018). Existence of vancomycin resistance among methicillin resistant *S. aureus* recovered from animal and human sources in Egypt. Slov. Vet. Res..

[29] Hanafy M., Hatem M. (1991). Studies on the antimicrobial activity of *Nigella sativa* seed (black cumin). J. Ethnopharmacol..

[30] Clinical and Laboratory Standards Institute Performance standards for Antimicrobial Susceptibility Testing, Twenty-First Informational Supplement CLSI Document M100-S21. Clinical and Laboratory Standards Institute, Wayne..

[31] Ahmed H.A., Tahoun A.B., Elez R.M.A., El-Hamid M.I.A., Ellatif S.S.A. (2019). Prevalence of *Yersinia enterocolitica* in milk and dairy products and the effects of storage temperatures on survival and virulence gene expression. Int. Dairy J..

[32] Reller L.B., Stratton C.W. (1977). Serum dilution test for bactericidal activity. II. Standardization and correlation with antimicrobial assays and susceptibility tests. J. Infect. Dis..

[33] Blas C.D., Wiseman J. (1998). CAB International, Wallingford Oxon (UK).

[34] Ibrahim D., Abdelfattah-Hassan A., Arisha A.H., El-Aziz R.M.A., Sherief W.R., Adil S.H., El Sayed R., Metwally A.E. (2020). Impact of feeding anaerobically fermented feed supplemented with acidifiers on its quality and growth performance, intestinal villi and enteric pathogens of mulard ducks. Livest. Sci..

[35] Ibrahim D., Sewid A.H., Arisha A.H., El-Fattah A.H.A., Abdelaziz A.M., Al-Jabr O.A., Kishawy A.T. (2020). Influence of glycyrrhiza glabra extract on growth, gene expression of gut integrity, and *Campylobacter jejuni* colonization in broiler chickens. Front. Vet. Sci..

[36] Farahat M., Ibrahim D., Kishawy A., Abdallah H., Hernandez-Santana A., Attia G. (2020). Effect of cereal type and plant extract addition on the growth performance, intestinal morphology, caecal microflora, and gut barriers gene expression of broiler chickens. Animal.

[37] Omar A.E., Al-Khalaifah H.S., Ismail T.A., El-Aziz R.M.A., El-Mandrawy S.A., Shalaby S.I., Ibrahim D. (2021). Performance, serum biochemical and immunological parameters, and digestive enzyme and intestinal barrier-related gene expression of broiler chickens fed fermented fava bean by-products as a substitute for conventional feed. Front. Vet. Sci..

[38] Blasco A., Ouhayoun J. (1996). Harmonization of criteria and terminology in rabbit meat research. Revised proposal. World Rabbit Sci..

[39] Livak K.J., Schmittgen T.D. (2001). Analysis of relative gene expression data using real-time quantitative PCR and the 2^−ΔΔCT^ method. Methods.

[40] Ibrahim D., Ismail T.A., Khalifa E., El-Kader A., Shaimaa A., Mohamed D.I., Mohamed D.T., Shahin S.E., El-Hamid A., Marwa I. (2021). Supplementing garlic nanohydrogel optimized growth, gastrointestinal integrity and economics and ameliorated necrotic enteritis in broiler chickens using a *Clostridium perfringens* challenge model. Animals.

[41] El-Hamid M.I.A., Ibrahim S.M., Eldemery F., El-Mandrawy S.A., Metwally A.S., Khalifa E., Elnahriry S.S., Ibrahim D. (2021). Dietary cinnamaldehyde nanoemulsion boosts growth and transcriptomes of antioxidant and immune related genes to fight Streptococcus agalactiae infection in Nile tilapia (*Oreochromis niloticus*). Fish Shellfish Immunol..

[42] Ibrahim D., Kishawy A.T., Khater S.I., Khalifa E., Ismail T.A., Mohammed H.A., Elnahriry S.S., Tolba H.A., Sherief W.R., Farag M.F. (2021). Interactive effects of dietary quercetin nanoparticles on growth, flesh antioxidant capacity and transcription of cytokines and Aeromonas hydrophila quorum sensing orchestrating genes in Nile tilapia (*Oreochromis niloticus*). Fish Shellfish Immunol..

[43] Ahmad A., Husain A., Mujeeb M., Khan S.A., Najmi A.K., Siddique N.A., Damanhouri Z.A., Anwar F. (2013). A review on therapeutic potential of *Nigella sativa*: A miracle herb. Asian Pac. J. Trop. Biomed..

[44] Hannan A., Saleem S., Chaudhary S., Barkaat M., Arshad M.U. (2008). Anti bacterial activity of *Nigella sativa* against clinical isolates of methicillin resistant *Staphylococcus aureus*. J. Ayub Med. Coll. Abbottabad.

[45] Hartmann M., Berditsch M., Hawecker J., Ardakani M.F., Gerthsen D., Ulrich A.S. (2010). Damage of the bacterial cell envelope by antimicrobial peptides gramicidin S and PGLa as revealed by transmission and scanning electron microscopy. Antimicrob. Agents Chemother..

[46] Toghyani M., Toghyani M., Gheisari A., Ghalamkari G., Mohammadrezaei M. (2010). Growth performance, serum biochemistry and blood hematology of broiler chicks fed different levels of black seed (*Nigella sativa*) and peppermint (Mentha piperita). Livest. Sci..

[47] Jamroz D., Kamel C. (2002). Plant extracts enhance broiler performance. In non-ruminant nutrition: Antimicrobial agents and plant extracts on immunity, health and performance. J. Anim. Sci..

[48] Kishawy A.T., Al-Khalaifah H.S., Nada H.S., Roushdy E.M., Zaglool A.W., Ahmed Ismail T., Ibrahim S.M., Ibrahim D. (2022). Black pepper or radish seed oils in a new combination of essential oils modulated broiler chickens’ performance and expression of digestive enzymes, lipogenesis, immunity, and autophagy-related genes. Vet. Sci..

[49] Al-Beitawi N., El-Ghousein S. (2008). Effect of feeding different levels of *Nigella sativa* seeds (black cumin) on performance, blood constituents and carcass characteristics of broiler chicks. Int. J. Poult. Sci..

[50] Hannan M.A., Rahman M.A., Sohag A.A.M., Uddin M.J., Dash R., Sikder M.H., Rahman M.S., Timalsina B., Munni Y.A., Sarker P.P. (2021). Black cumin (*Nigella sativa* L.): A comprehensive review on phytochemistry, health benefits, molecular pharmacology, and safety. Nutrients.

[51] Asghar M.U., Doğan S.C., Wilk M., Korczyński M. (2022). Effect of dietary supplementation of black cumin seeds (*Nigella sativa*) on performance, carcass traits, and meat quality of Japanese quails (Coturnix coturnix japonica). Animals.

[52] Tabler T.W., Greene E.S., Orlowski S.K., Hiltz J.Z., Anthony N.B., Dridi S. (2020). Intestinal barrier integrity in heat-stressed modern broilers and their ancestor wild jungle fowl. Front. Vet. Sci..

[53] Ibrahim D., Abdelfattah-Hassan A., Badawi M., Ismail T.A., Bendary M.M., Abdelaziz A.M., Mosbah R.A., Mohamed D.I., Arisha A.H., El-Hamid M.I.A. (2021). Thymol nanoemulsion promoted broiler chicken’s growth, gastrointestinal barrier and bacterial community and conferred protection against *Salmonella* Typhimurium. Sci. Rep..

[54] Kapan M., Tekin R., Onder A., Firat U., Evliyaoglu O., Taskesen F., Arikanoglu Z. (2012). Thymoquinone ameliorates bacterial translocation and inflammatory response in rats with intestinal obstruction. Int. J. Surg..

[55] Patra A.K. (2020). Influence of plant bioactive compounds on intestinal epithelial barrier in poultry. Mini Rev. Med. Chem..

[56] Patra A.K., Amasheh S., Aschenbach J.R. (2019). Modulation of gastrointestinal barrier and nutrient transport function in farm animals by natural plant bioactive compounds–a comprehensive review. Crit. Rev. Food Sci. Nutr..

[57] Salem M.L. (2005). Immunomodulatory and therapeutic properties of the *Nigella sativa* L. seed. Int. Immunopharmacol..

[58] Al-Mufarrej S. (2014). Immune-responsiveness and performance of broiler chickens fed black cumin (*Nigella sativa* L.) powder. J. Saudi Soc. Agric. Sci..

[59] El-Bagir N.M., Farah I.T., Elhag S.M., Alhaidary A., Mohamed H.E., Beynen A.C. (2010). Immune response and pasteurella resistance in rabbits fed diets containing various amounts of black cumin seeds. Am. J. Anim. Vet. Sci..

[60] Mahmoud H.S., Almallah A.A., EL-Hak H.N.G., Aldayel T.S., Abdelrazek H., Khaled H.E. (2021). The effect of dietary supplementation with *Nigella sativa* (black seeds) mediates immunological function in male Wistar rats. Sci. Rep..

[61] Amin B., Hosseinzadeh H. (2016). Black cumin (Nigella sativa) and its active constituent, thymoquinone: An overview on the analgesic and anti-inflammatory effects. Planta Med..

[62] Zaoui A., Cherrah Y., Alaoui K., Mahassine N., Amarouch H., Hassar M. (2002). Effects of *Nigella sativa* fixed oil on blood homeostasis in rat. J. Ethnopharmacol..

[63] Fararh K., Atoji Y., Shimizu Y., Shiina T., Nikami H., Takewaki T. (2004). Mechanisms of the hypoglycaemic and immunopotentiating effects of *Nigella sativa* L. oil in streptozotocin-induced diabetic hamsters. Res. Vet. Sci..

[64] Haq A., Lobo P.I., Al-Tufail M., Rama N.R., Al-Sedairy S.T. (1999). Immunomodulatory effect of *Nigella sativa* proteins fractionated by ion exchange chromatography. Int. J. Immunopharmacol..

[65] Elmowalid G., Amar A.M., Ahmad A.A.M. (2013). *Nigella sativa* seed extract: 1. Enhancement of sheep macrophage immune functions in vitro. Res. Vet. Sci..

[66] Majdalawieh A.F., Hmaidan R., Carr R.I. (2010). *Nigella sativa* modulates splenocyte proliferation, Th1/Th2 cytokine profile, macrophage function and NK anti-tumor activity. J. Ethnopharmacol..

[67] Salem M.L., Hossain M.S. (2000). Protective effect of black seed oil from *Nigella sativa* against murine cytomegalovirus infection. Int. J. Immunopharmacol..

[68] Boskabady M.-H., Keyhanmanesh R., Khameneh S., Doostdar Y., Khakzad M.-R. (2011). Potential immunomodulation effect of the extract of *Nigella sativa* on ovalbumin sensitized guinea pigs. J. Zhejiang Univ. Sci. B.

