# Supplementary material for: Nigella sativa Extract Potentially Inhibited Methicillin Resistant Staphylococcus aureus Induced Infection in Rabbits: Potential Immunomodulatory and Growth Promoting Properties
Source: Animals (Basel). 2022 Sep 30;12(19):2635. doi: 10.3390/ani12192635 (PMC9559630; doi:10.3390/ani12192635)
Supplement: Supplementary file 1 [file animals-12-02635-s001.zip › animals-1926407-supplementary.pdf]

Supplementary Table S1. Antimicrobial resistance patterns of MRSA isolates from animal and human origins

Antimicrobial/resistance pattern	No. of MRSA isolates from different origins (%)		Total (23)
	Animal (9)	Human (14)	
Methicillin	9 (100)	14 (100)	23 (100)
Erythromycin	2 (22.2)	6 (42.9)	8 (34.8)
Doxycycline	3 (33.3)	5 (35.7)	8 (34.8)
Clindamycin	2 (22.2)	4 (28.6)	6 (26.1)
Amoxicillin/clavulanic acid	6 (66.7)	5 (35.7)	11 (47.8)
Sulfamethoxazole/trimethoprim	1 (11.1)	8 (57.1)	9 (39.1)
Ciprofloxacin	3 (33.3)	5 (35.7)	8 (34.8)
Rifampicin	2 (22.2)	3 (21.4)	5 (21.7)
Gentamicin	0 (0)	1 (7.1)	1 (4.3)
Multidrug resistance pattern	5 (55.6)	8 (57.1)	13 (56.5)
